# Heterocyclic Amine-Induced Feeding Deterrence and Antennal Response of Honey Bees

**DOI:** 10.3390/insects12010069

**Published:** 2021-01-14

**Authors:** Nicholas R. Larson, Scott T. O’Neal, Thomas P. Kuhar, Ulrich R. Bernier, Jeffrey R. Bloomquist, Troy D. Anderson

**Affiliations:** 1 Department of Entomology, Virginia Tech, Blacksburg, VA 24061, USA; nicholas.larson@usda.gov (N.R.L.); tkuhar@vt.edu (T.P.K.); 2 Department of Entomology, University of Nebraska, Lincoln, NE 68588, USA; onealst@vt.edu; 3 USDA Agricultural Research Service, Center for Medical, Agricultural and Veterinary Entomology, Gainesville, FL 32608, USA; Uli.Bernier@ARS.USDA.GOV; 4 Emerging Pathogens Institute, Department of Entomology and Nematology, University of Florida, Gainesville, FL 32611, USA; jbquist@epi.ufl.edu

**Keywords:** honey bee, heterocyclic amines, repellent, behavior, feeding deterrence

## Abstract

**Simple Summary:**

This study examined the behavioral and antennal effects of heterocyclic amines (HCAs) on forager honey bees. Behavioral changes related to feeding were initially characterized using a video-tracking protocol in which individual foragers were exposed to HCA-treated food sources within a Petri dish arena. The most efficacious HCA was then tested in a field study using a high-tunnel arena to determine whether repellent effects could be observed on a larger number of foragers to a treated food source. The same HCA was then tested in the field on both melon flowers and knapweed bundles to observe whether repellency was conserved in a more agriculturally realistic scenario. Finally, electroantennogram (EAG) experiments were conducted to document whether the honey bee olfactory system was detecting these compounds. These findings suggest that HCAs could provide an active approach to deter honey bee foragers from feeding on treated agricultural crops.

**Abstract:**

The productivity and survival of managed honey bee colonies is negatively impacted by a diverse array of interacting factors, including exposure to agrochemicals, such as pesticides. This study investigated the use of volatile heterocyclic amine (HCA) compounds as potential short-term repellents that could be employed as feeding deterrents to reduce the exposure of bees to pesticide-treated plants. Parent and substituted HCAs were screened for efficacy relative to the repellent *N*,*N*-diethyl-meta-toluamide (DEET) in laboratory and field experiments. Additionally, electroantennogram (EAG) recordings were conducted to determine the level of antennal response in bees. In video-tracking recordings, bees were observed to spend significantly less time with an HCA-treated food source than an untreated source. In a high-tunnel experiment, the HCA piperidine was incorporated in a feeding station and found to significantly reduce bee visitations relative to an untreated feeder. In field experiments, bee visitations were significantly reduced on melon flowers (*Cucumis melo* L.) and flowering knapweed (*Centaurea stoebe* L.) that were sprayed with a piperidine solution, relative to untreated plants. In EAG recordings, the HCAs elicited antennal responses that were significantly different from control or vehicle responses. Overall, this study provides evidence that HCAs can deter individual bees from food sources and suggests that this deterrence is the result of antennal olfactory detection. These findings warrant further study into structure–activity relationships that could lead to the development of short-term repellent compounds that are effective deterrents to reduce the contact of bees to pesticide-treated plants.

## 1. Introduction

Insect pollination services are estimated to provide $20 billion in annual benefits to the agricultural sectors in both Europe and North America, with the annual benefits to agriculture globally estimated at $212 billion [1]. However, pesticide exposure from the widespread use of agrochemicals in food production can have deleterious effects on honey bee colonies [2,3]. A recent risk assessment found that 161 pesticide residues have been identified in bee hives across the world, of which insecticides comprised 52%, fungicides 25%, herbicides 17%, and acaricides 6% [4]. Exposure to pesticides can produce effects in bees that range from acutely toxic to sublethal, including disorientation, paralysis, and behavioral changes [3], as well as altered development and reduced immunocompetence [5,6,7,8]. Nonetheless, it remains likely that pesticides will continue to be used for the foreseeable future, as they remain critical for achieving high yields in modern agriculture [9]. To mitigate harm to honey bees, there is a need for novel approaches and strategies to limit pollinator exposure to pesticides and other agrochemicals [2]. One strategy that has been investigated since the early 1950s is the co-application of repellent compounds to blooming plants that have been treated with pesticides in order to minimize contact between pollinators and agrochemicals [10,11,12].

**Figure 1:**
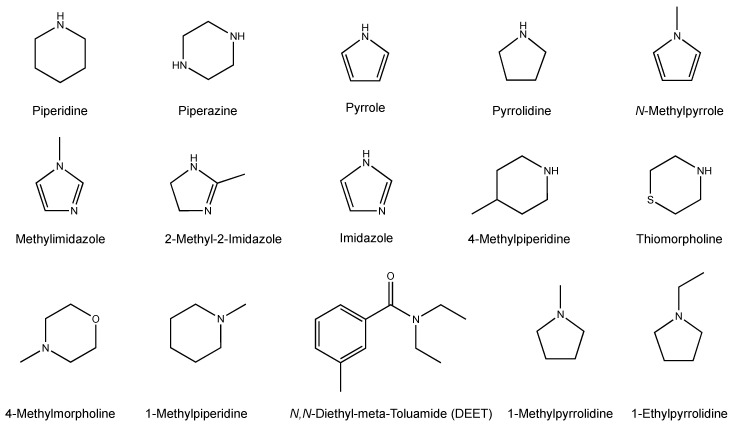
Chemical structures of parent heterocyclic amines, substituted pyrroles, substituted piperidines and DEET. Parent compounds: piperidine, piperazine, pyrrole, and pyrrolidine. Substituted pyrroles: *N*-methylpyrrole, 2-methylimidazole, 2-methyl-2-imidazoline, and imidazole. Substituted piperidines: 4-methylpiperidine, thiomorpholine, 4-methylmorpholine, and 1-methylpiperidine. Substituted pyrrolidines: 1-methylpyrroldine and 1-ethylpyrrolidine. DEET: *N*,*N*-diethyl-*meta*-toluamide.

Historically, insect repellents have been used to reduce the spread of deadly pathogens by arthropod vectors [13]. Before World War II and the introduction of *N*,*N*-diethyl-*meta*-toluamide (DEET, Figure 1), only four insect repellents were available for consumer use, including citronella oil, dialkyl phthalate, Indalone^®^, and Rutgers 612 [14]. Since its commercial introduction in 1956, DEET has remained the most efficacious and widely used insect repellent [13,14,15], and it is recommended by both the World Health Organization and the United States Environmental Protection Agency (EPA) as a positive control for repellent screening [16]. However, concerns have arisen regarding the safety of DEET, which has led to an ongoing search for safer repellent chemistries [17]. One such group of chemistries is the family of synthetic compounds related to piperidine, which have been evaluated for their potential use as repellents [18,19,20,21,22].

Piperidine is a heterocyclic amine (HCA) consisting of a six-membered ring structure with five carbons and one nitrogen (Figure 1). The analog 1-(3-cyclohexen-1-ylcarbonyl)-2-methylpiperidine has been shown to repel the lone star tick, *Amblyomma americanum*, from humans significantly longer than DEET, and one of its stereoisomers has been reported as an effective repellent for mosquitoes and chigger mites [19,21]. Similarly, 1-(3-cyclohexenyl-carbonyl) piperidine was demonstrated to provide a longer protection time against the lone star tick than DEET [23]. The EPA currently recognizes seven active ingredients for topically applied insect repellents, including picaridin, a piperidine compound shown to be a highly effective tick repellent. The use of HCAs as a novel method for inhibiting host finding in mosquitoes has also been proposed, as they have been hypothesized to cause hyposmic or anosmic effects in mosquitoes [24], although recent studies have shown that mosquito antennae do respond to these compounds [25].

This study examined the behavioral and antennal effects of HCAs on forager honey bees. Behavioral changes related to feeding were initially characterized using a video-tracking protocol in which individual foragers were exposed to HCA-treated food sources within a Petri dish arena. The most efficacious HCA was then tested in a field study using a high-tunnel arena to determine whether repellent effects could be observed on a larger number of foragers to a treated food source. The same HCA was then tested in the field on both melon flowers and knapweed bundles to observe whether repellency was conserved in a more agriculturally realistic scenario. Finally, electroantennogram (EAG) experiments were conducted to document whether the honey bee olfactory system was detecting these compounds. These findings suggest that HCAs could provide an active approach to deter honey bee foragers from feeding on treated agricultural crops.

## 2. Materials and Methods

### 2.1. Subjects

Research subjects for all laboratory and field experiments consisted of European honey bees (*Apis mellifera* carnica). For the video-tracking recordings, bees were obtained from established colonies maintained by the Department of Entomology at Virginia Tech (Blacksburg, VA, USA). In order to reduce the likelihood of collecting newly emerged bees, workers were collected from the top super of each colony and then housed in a laboratory incubator at 32 °C with a relative humidity of 65 to 75%. Food was withheld overnight prior to testing in behavioral assays. For the EAG recordings, bees were obtained from established colonies maintained by the Department of Entomology at the University of Nebraska (Lincoln, NE, USA). Workers were collected from the top super of each colony and then housed in a laboratory incubator at 32 °C with a relative humidity of 65 to 75%. The bees were cold anaesthetized for experimental use.

### 2.2. Chemicals

**Figure 2:**
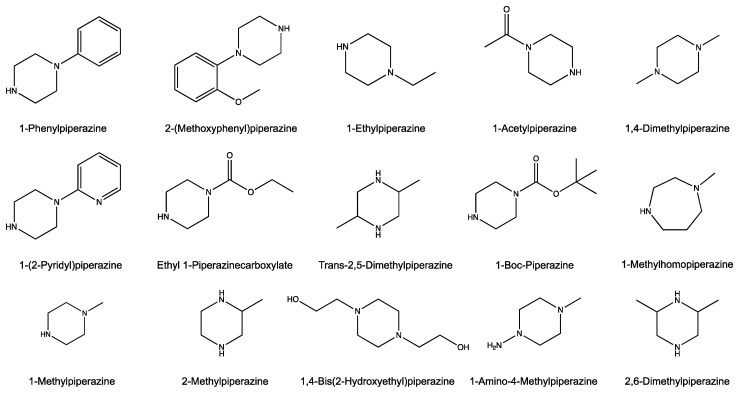
Chemical structure of substituted piperazines. Substituted piperazines: 1-phenylpiperazine, 2-(methoxyphenyl)piperazine, 1-ethylpiperazine, 1-acetylpiperazine, 1,4-dimethylpiperazine, 1-(2-pyridyl)piperazine, ethyl-1-piperazinecarboxylate, trans-2,5-dimethylpiperazine, 1-boc-piperazine, 1-methylhomopiperazine, 1-methylpiperazine, 2-methylpiperazine, 1,4-bis(2-hydroxyethyl)piperazine, 1-amino-4-methylpiperazine, and 2,6-dimethylpiperazine.

The heterocyclic amines (Figure 1 and Figure 2) were purchased at the highest available purity from Sigma-Aldrich Chemical Co. (St. Louis, MO, USA). OFF! Deep Woods^®^ Sportsmen (SC Johnson, Racine, WI, USA) was used as the source for DEET (98% active ingredient; *N*,*N*-diethyl-*meta*-toluamide).

### 2.3. Video-Tracking Recordings

Changes in honey bee feeding behavior were observed using a video-tracking protocol as previously described [26]. Behavior was recorded using EthoVision XT video recording software and a Basler acA-1300-60gm camera (Noldus, Leesburg, VA, USA). The recording arena was draped in a black plastic tarp to block ambient light, and illuminated solely with light in the red spectrum, to avoid inducing light bias, using Cryon LED lights (Model HTP904E, Chatsworth, CA, USA). HCA compounds were evaluated, relative to untreated controls and DEET, for their ability to deter feeding when suspended in 1.5 × 1.5 × 0.3 cm sugar-agarose cube. Sugar-agarose cubes were prepared by combining 8 g of sucrose, 20 mL deionized water, and 170 mg agarose, then adding DEET and HCAs to yield a final concentration of 10 µL/mL within the solution (controls were left untreated), before finally transferring aliquots of the solution to individual molds and allowing each to set.

For behavioral video-tracking experiments, standard, transparent plastic Petri dishes were arranged beneath the camera in a 4 × 4 grid pattern. A sugar-agarose cube was placed in the center of each dish, along with a single bee, and then covered with a lid that had holes drilled it for ventilation. Eight treated and untreated sugar-agarose cubes were tested each time, and bee movement was recorded and tracked for 10 min. The amount of time spent by each bee in the area of the sugar-agarose cube, designated the feeding zone, was calculated using EthoVision video-tracking software (Noldus Information Technology, Wageningen, the Netherlands). The differences between each treatment group were statistically analyzed using one-way ANOVA with Tukey’s post-hoc test (GraphPad Prism 8.3.0, La Jolla, CA, USA).

### 2.4. High-Tunnel Experiments

**Figure 3:**
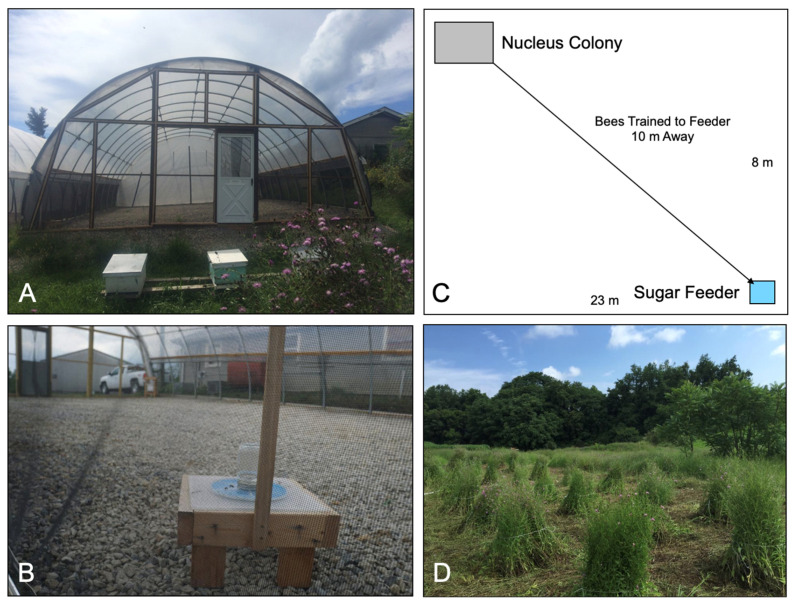
Graphical representation of high-tunnel and knapweed field experiments. Two nucleus colonies were placed into the corner of identical high tunnels (**A**) and the bees were trained to sugar feeders (**B**) located 10 m from the opposite corners of the tunnels (**C**) for 9 days. The sugar feeders were replaced with a sugar solution containing 10 µL/mL piperidine on day 10. Visitation to the feeders was then recorded at 5 min intervals for 1 h using cameras. (**D**) Knapweed bundles sprayed with H_2_O, DEET, or piperidine.

A previously described high-tunnel protocol was used to evaluate the number of bees that visited a feeder station [27], which was either treated with the HCA piperidine or left untreated (Figure 3A–C). Two queenright, nucleus bee colonies were established by splitting larger colonies from the Kentland Farms apiary of Virginia Tech in Whitethorne, VA, USA. These nucleus colonies were moved to the Price’s Fork Research Center of Virginia Tech (near Blacksburg, VA, USA) where each colony was placed into a separate high tunnel. The high-tunnel dimensions are 10.97 m L × 7.92 m W × 3.66 m H. The bees from each nucleus colony were trained to a feeding station containing 50% (*w*/*v*) sucrose solution for 2 d. The feeding stations were placed in front of the hive in the morning for a 2 h period. After 2 h, the sucrose solution was replaced with water only and left in the same location overnight. The next morning, the water was replaced with the sucrose solution and the feeding stations were moved 10 m from the hive. Once the bees were trained to the feeding stations, 10 µL/mL piperidine (HCA treatment) was dissolved in 50% sucrose for one of the feeding stations, while the other feeding station contained a 50% sucrose solution only (control treatment). A video camera (GoPro, Inc., San Mateo, CA, USA) was used to record the number of bees visiting the control and piperidine-treated feeding stations for a 2 h period. The video cameras were programmed to take pictures of the visiting bees at 1 min intervals. The video recordings were analyzed at 5 min intervals for 60 min using ImageJ software (National Institutes of Health, Bethesda, MD, USA). Each experiment was repeated in duplicate with two colonies per treatment. Linear regression analysis was used to analyze each treatment with GraphPad Prism software (La Jolla, CA, USA).

### 2.5. Field Experiments with Melon Flowers and Knapweed

A nucleus bee colony was moved from the Moore Farm of Virginia Tech (near Blacksburg, VA) to ‘Athena’ melon (*Cucumis melo* L.) plots at Virginia Tech’s Kentland Farm. The melon plants were arranged in 5 plots with 4 rows per plot and approximately 1.5 m between each plot. Three observers were assigned to each plot and each observer was assigned one row. Observers were randomly assigned a 1000 mL spray bottle containing 250 mL deionized H_2_O (control treatment), 10 µL/mL DEET (positive control), or 10 µL/mL piperidine (HCA treatment). Each observer positioned themselves at 20 melon flowers and recorded the number of bees visiting the untreated flowers for a 3 min period. A bee visit was recorded as one individual landing and actively foraging on the flower. Those bees that landed on and immediately left the flower were not reported as a visitation. Melon flower pollen is less desirable to honey bees than other pollen, so in addition to honey bees, the number of squash bees, *Peponapis pruinosa* Say, visiting the flowers was also recorded by each observer. After the initial 3 min observation period, an observer delivered the treatments to each of the flowers with a single pump (~2 mL) of the spray bottle assigned to the observer. A second 3 min observation period was conducted and the number of bees visiting the flowers was recorded following the application of treatments. The spray bottles containing the treatments were randomly assigned again, and the same method was applied to the next melon plot. The experiment was performed over 2 d with a total of 12 pre- and post-spray observations per treatment. The pre- and post-number of bees visiting the treated melon flowers was statistically analyzed using an ANCOVA with JMP (SAS, Cary, NC, USA). The pre-treatment bee visits were used as the covariate and the ANCOVA was valid if the interaction between the treatment and covariate was non-significant (*p* < 0.05). Student’s *t*-test was used to determine the significance between the post-visitation of bees to the treatments. 

A small field plot of blooming knapweed (*Centaurea stoebe* L.), located at the Prices Fork Research Center apiary, was divided into 30 bundles of 1 × 1 m in size with approximately 1 m of space between bundles (Figure 3D). Three observers were each assigned to a bundle and randomly assigned a 1000 mL spray bottle containing 250 mL deionized H_2_O (negative control), 10 µL/mL DEET (positive control), or 10 µL/mL piperidine (HCA treatment). Each observer positioned themselves near their assigned bundle and recorded the number of bees visiting the untreated bundle for a 3 min period. A bee visit was recorded as one individual landing and actively foraging on the flower. Those bees that landed on and immediately left the flower were not reported as a visitation. After the initial 3 min observation period, an observer delivered the treatment to the bundle, spraying to saturation (approximately 20–25 pumps) with the spray bottle assigned. A second 3 min observation period was conducted and the number of bees visiting the bundles was recorded following the application of treatments. The spray bottles containing the treatments were randomly assigned again, and same method was applied to the next set of knapweed bundles. The experiment was performed over 3 d with a total of 17 pre- and post-spray observations. The pre- and post-number of bees visiting the treated knapweed bundles was statistically analyzed using an ANCOVA with JMP (SAS, Cary, NC, USA). The pre-treatment bee visits were used as the covariate and the ANCOVA was valid if the interaction between the treatment and covariate was non-significant (*p* < 0.05). Student’s *t*-test was used to determine the significance between the post-visitation of bees to the treatments.

### 2.6. Electroantennogram (EAG) Recordings

EAG recordings were conducted as previously described [28,29]. Chemicals were diluted into a hexane (Sigma-Aldrich, St. Louis, MO, USA) carrier solvent at 10, 50, 100, 500, 1000 µL/mL and stored at 4 °C prior to the EAG recordings. For the concentration response bioassays, a serial dilution of each chemical was prepared using a hexane carrier solvent. A 10 µL aliquot of each chemical was applied to a strip of Whatman No. 1 filter paper (Sigma-Aldrich, St. Louis, MO, USA) and then allowed to dry for 30–60 sec before inserting the chemical-treated paper into a Pasteur pipette (15 cm long). EAG recordings were conducted using Ag–AgCl electrodes inserted into glass capillary tubes filled with electrogel (Spectro 360, Parker Laboratory, NJ, USA). Thin-walled glass capillary tubes (World Precision Instruments, Sarasota, FL, USA) were pulled to a fine tip with a P-97 Flaming/Brown Micropipette puller (Sutter Instrument, Novato, CA, USA) using the following settings: Heat = 500; Pull = 25; Velocity = 200; Time = 500; Pressure = 500. Bee antennae were randomly excised from cold anesthetized individuals below the scape with microscissors under a dissection microscope. The glass capillary tube was cut at the tip with microforceps to fit the base of a bee antenna, while the other end of the capillary tube was attached to a reference electrode. The reference electrode was then positioned so that the tip of the excised antenna was near the recording electrode. A second glass capillary tube was prepared as described above to fit the cut tip of the bee antenna, and the other end of the capillary tube was attached to a recording electrode. The recording electrode was connected to a high-impedance D.C. amplifier (IDAC 4 Ockenfels Syntech Gmbh, Kirchzarten, Germany) and change in baseline DC voltage (mV) was analyzed using EAG Pro 1.1 software (Syntech, Stilwell, KS, USA). 

A bee antenna was exposed to a compound by inserting a Pasteur pipette containing a chemical-treated filter paper into the opening of a glass mixing tube that was positioned perpendicular to the suspended bee antenna. The mixing tube provided a constant flow of humidified air over the prepared bee antenna. A 1 sec puff of air was passed through the Pasteur pipette and into the mixing tube to deliver the chemical to the bee antenna. The signal was allowed to return to the baseline (*ca.* 30–90 sec) before the application of another stimulus. An empty Pasteur pipette and hexane- or ethanol-treated filter paper served as a control and carrier solvent treatment, respectively. A total of 2 to 7 bee antennae were exposed to the compounds in a randomized order. The baseline voltage output of the bee antennae was determined prior to compound exposure, and the antennae were allowed to return to the baseline voltage before applying the next compound. For the concentration-response bioassays, a new antenna was used for each concentration (*n* ≥ 3). A lowest detection point was determined by the program, indicating the voltage at the lowest point after a stimulus was applied. The absolute values of these two points for each stimulus were calculated and the baseline value was subtracted from the lowest detection point to give the change in baseline voltage (mV). The differences between each treatment group were statistically analyzed using one-way ANOVA with Tukey’s post-hoc test (GraphPad Prism 8.3.0, La Jolla, CA, USA).

## 3. Results

### 3.1. Video-Tracking Recordings

**Figure 4:**
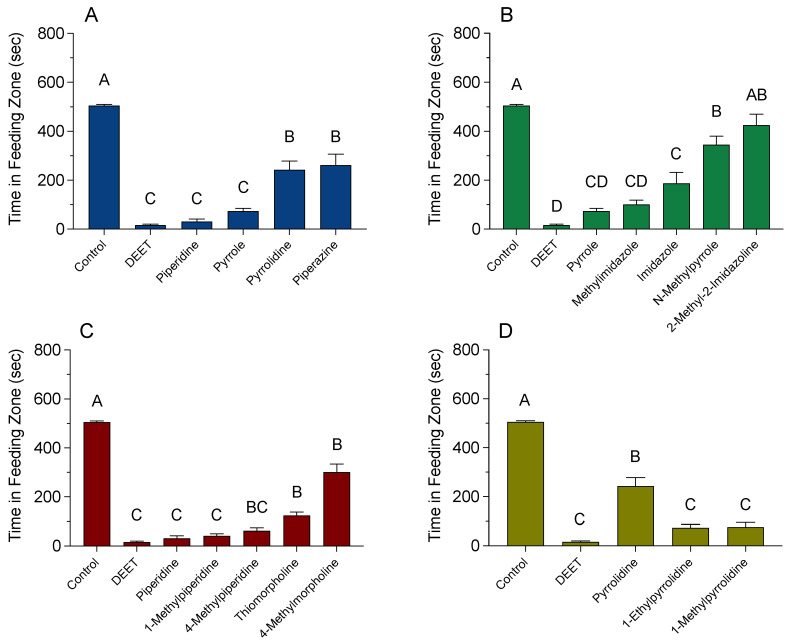
Effect of heterocyclic amines (HCAs) on the feeding behavior of honey bees. A video-tracking protocol to screen individual bees for behavior changes when exposed to sugar-agarose cubes treated with HCAs (10 µL/mL). Controls were sugar-agarose cubes with no treatment. DEET-treated sugar-agarose cubes were screened in each experimental cohort as a positive control. (**A**) HCA parent compounds (F_(5, 488)_ = 173.70, *p* < 0.0001). (**B**) Substituted pyrroles (F_(6, 523)_ = 123.10, *p* < 0.0001). (**C**) Substituted piperidines (F_(6, 504)_ = 221.30, *p* < 0.0001). (**D**) Substituted pyrrolidines (F_(4, 479)_ = 221.20, *p* < 0.0001). Vertical bars represent the mean ± standard error. Different letters above bars indicate significant differences using one-way ANOVA with Tukey’s post-hoc test (*p* < 0.05).

The effects of HCAs and substituted HCAs at 10 µL/mL on the feeding behavior of honey bees are shown in Figure 4A–D. The amount of time spent by bees in the feeding zones with HCA- or DEET-treated sugar-agarose cubes had the following rank order of effectiveness based on statistical significance: control (more time spent) > piperazine = pyrrolidine > pyrrole = piperidine = DEET (less time spent) (Figure 4A). Bees were recorded in the feeding zone of the untreated control sugar-agarose cubes for 505 ± 5 sec, whereas bees exposed to the DEET-treated sugar-agarose cubes were in the feeding zone for 16 ± 4 sec. Piperidine and pyrrole reduced the time bees spent in the feeding zone significantly more than that of the other HCAs. Bees exposed to piperidine-treated cubes were in the feeding zone for 31 ± 10 sec and bees exposed to pyrrole-treated cubes spent 74 ± 12 sec in the feeding zone. Pyrrolidine and piperazine were less effective at reducing the time spent by bees in the feeding zone. Bees exposed to pyrrolidine-treated cubes were in the feeding zone for 243 ± 35 sec, whereas bees exposed to piperazine-treated cubes spent 262 ± 45 sec in the feeding zone.

A different structure of pyrrole reduced the amount of time that bees spent in the feeding zone (Figure 4B). The amount of time spent by bees in the feeding zones with substituted pyrrole-treated sugar-agarose cubes had the following rank order of effectiveness based on statistical significance: control (more time spent) = 2-methyl-2-imidazoline = *N*-methylpyrrole > imidazole = methyl imidazole = pyrrole > DEET (less time spent). Bees exposed to methyl imidazole-treated cubes spent 101 ± 17 sec in the feeding zone, which was 27 sec longer than the feeding zone time spent by bees exposed to pyrrole-treated cubes. Similarly, bees exposed to imidazole-treated cubes were present in the feeding zone for 187 ± 45 sec, which is 113 sec longer than that of bees exposed to pyrrole-treated cubes. However, there was no significant difference between the time bees spent in the feeding zone with methyl imidazole-treated cubes when compared to those with imidazole-treated cubes. Interestingly, *N*-methylpyrrole treatment significantly increases the time spent by the bees in feeding zone (345 ± 35 s) when compared to bees exposed to pyrrole treatment. Bees exposed to 2-methyl-2-imidazoline-treated cubes spent 425 ± 45 sec in the feeding zone, which is comparable to the bees provided with untreated cubes.

A different structure of piperidine also significantly decreased the amount of time that bees spent in the feeding zone (Figure 4C). The amount of time spent by bees in the feeding zones with substituted piperidine-treated sugar-agarose cubes had the following rank order of effectiveness based on statistical significance: control (more time spent) > 4-methylmorpholine = thiomorpholine = 4-methylpiperidine > 1-methylpiperidine = piperidine = DEET (less time spent). Bees exposed to cubes treated with 1-methylpiperidine and thiomorpholine spent 42 ± 8 sec and 124 ± 14 sec in the feeding zone, respectively, and did not differ significantly from exposure to either piperidine- or DEET-treated cubes. In addition, 4-methylmorpholine was the least effective substituted piperidine. Bees exposed to 4-methylmorpholine-treated cubes were present in the feeding zone for 301 ± 33 sec, which is 270 sec longer than bees exposed to piperidine-treated agarose cubes.

The amount of time spent by bees in the feeding zones with substituted pyrrolidine-treated sugar-agarose cubes had the following rank order of effectiveness based on statistical significance: control (more time spent) > pyrrolidine > 1-ethylpyrrolidine = 1-methylpyrrolidine = DEET (less time spent) (Figure 4D). Bees exposed to cubes treated with 1-ethylpyrrolidine and 1-methylpyrrolidine spent 73 ± 14 sec and 76 ± 19 sec in the feeding zone, respectively, which was significantly less time than bees exposed to pyrrolidine-treated cubes spent in the feeding zone, but not significantly different from the time spent in the feeding zone by bees exposed to DEET-treated cubes.

**Figure 5:**
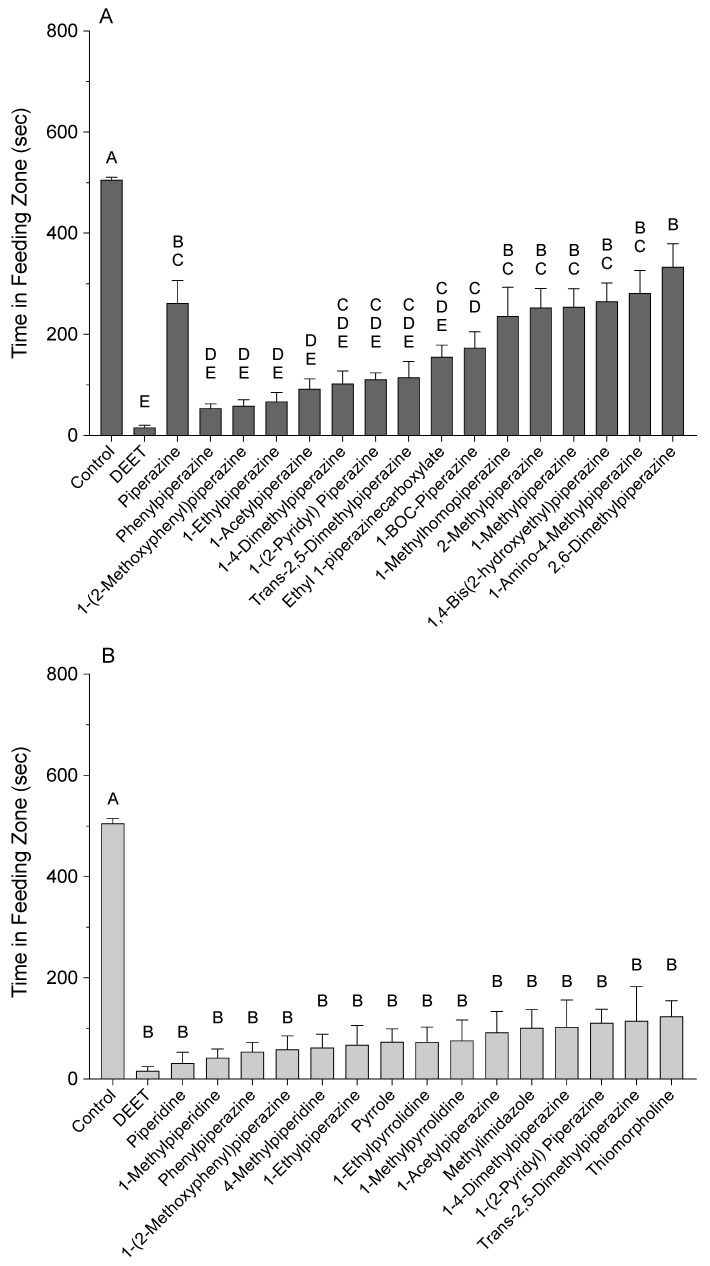
Effect of substituted piperazines on the feeding behavior of honey bees and summary plot of compounds with repellent activity statistically indistinguishable from DEET. A video-tracking protocol was used to screen individual bees in Petri dishes for behavior changes when exposed to sugar-agarose cubes treated with HCAs (10 µL/mL). Controls were sugar-agarose cubes with no treatment. DEET-treated sugar-agarose cubes were screened as a positive control. (**A**) Substituted piperazines. (**B**) Combined graph of HCAs that had deterrent effects similar to DEET. Vertical bars represent the mean ± standard error. Different letters above bars indicate significant differences using one-way ANOVA with Tukey’s post-hoc test (*p* < 0.05).

The amount of time spent in the feeding zones with substituted piperazine-treated sugar-agarose cubes had the following rank order of effectiveness based on statistical significance: control (more time spent) > 2,6-dimethylpiperazine > 1-amino-4-methylpiperazine > 1,4-bis(2-hydroxyethyl)piperazine = piperazine = 1-methylpiperazine = 2-methylpiperazine > 1-methylhomopiperzine > 1-boc-piperazine > ethyl 1-piperazinecarboxylate > trans-2,5-diemthylpiperazine = 1-(2-pyridyl) piperazine = 1-4-dimethylpiperazine > 1-acetylpiperazine = 1-ethylpiperazine = 1-(2-methoxyphenyl) piperazine = phenylpiperazine > DEET (less time spent) (Figure 5A). Bees exposed to phenylpiperazine-, 1-(2-methoxyphenyl) piperazine-, 1-ethylpiperazine-, and 1-acetylpiperazine-treated cubes spent 54 ± 9 sec, 59 ± 12 sec, 67 ± 18 sec, and 92 ± 19 sec in the feeding zone, respectively, which was significantly less than bees exposed to piperazine-treated cubes, but not bees exposed to DEET-treated cubes. Bees exposed to cubes treated with 1-4-dimethylpiperazine, 1-(2-pyridyl) piperazine, *trans*-2,5-diemthylpiperazine, and ethyl 1-piperazinecarboxylate spent 103 ± 25 sec, 111 ± 13 sec, 115 ± 32 sec, and 156 ± 23 sec in the feeding zone, respectively, which was not significantly different from bees exposed to piperazine- or DEET-treated cubes. Bees exposed to cubes treated with 1-boc-piperazine, 1-methylhomopiperzine, 2-methylpiperazine, and 1-methylpiperazine spent 174 ± 32 sec, 236 ± 57 sec, 253 ± 38 sec, and 254 ± 36 sec in the feeding zone, respectively, which was not significantly different from bees exposed to piperazine-treated cubes, but was significantly more time than bees exposed to DEET-treated cubes spent in the feeding zone. Bees exposed to cubes treated with 1,4-bis(2-hydroxyethyl) piperazine, 1-amino-4-methylpiperazine, and 2,6-dimethylpiperazine spent 265 ± 37 sec, 281 ± 45 sec, and 334 ± 46 sec in the feeding zone, respectively, which was not significantly different from bees exposed to piperazine-treated cubes, but was significantly more time than bees exposed to DEET-treated cubes spent in the feeding zone.

A summary plot of HCAs with repellent activity statistically indistinguishable from DEET is shown in Figure 5B, which includes at least one compound from each parental HCA structure that had effects that were not significantly different from DEET.

### 3.2. High-Tunnel Experiments

**Figure 6:**
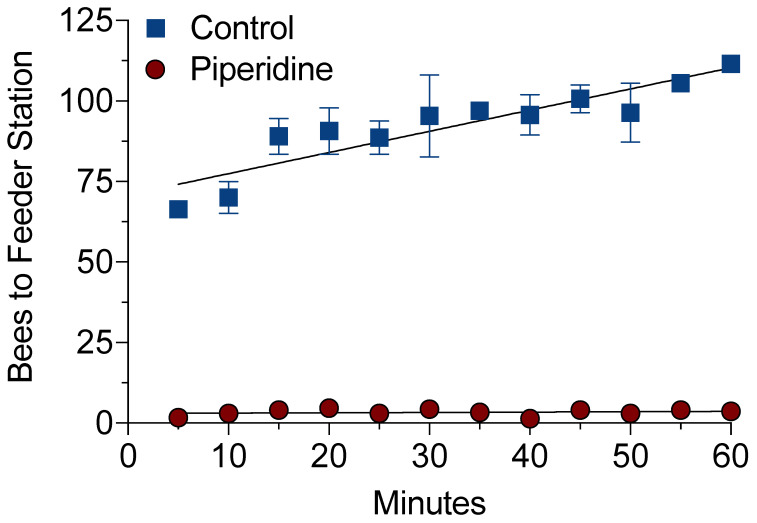
Number of honey bees visiting a feeder station treated with piperidine. Two high tunnels contained two nucleus bee colonies that were trained to a feeder station containing 50% (*w*/*v*) sucrose solution for 9 d. The sucrose solution was replaced at the feeder station with 10 µL/mL piperidine in a sucrose solution on day 10. The number of bees visiting the piperidine-untreated (control) and -treated feeder stations was video recorded for 60 min. Data were analyzed using linear regression analysis. Symbols represent the mean ± standard error and when absent the error bars are within the size of the symbol. Control equation: Y = 0.6578 * X + 70.85, *R*^2^ = 0.53. Treatment equation: Y = 0.01026 * X + 3, *R*^2^ = 0.005.

The high-tunnel experiments were conducted to examine the effect of piperidine on the number of bees visiting a feeding station that provided a sucrose-based food source (Figure 6). The maximum number of bees visiting the control (sucrose solution only) feeding station was 114 total individuals after a 60 min period. The maximum number of bees visiting the treated (10 µL/mL piperidine in sucrose solution) feeding station was 9 total individuals after a 60 min period. The average number of bees visiting a control feeder over the 60 min period was 91 ± 2.6, compared to the treated feeder with an average of 3 ± 0.4. A steady recruitment of bees was observed visiting the control feeding station (y = 0.6576x + 0.0952, *R*^2^ = 0.83), whereas fewer bees visited the treated feeding station (y = 0.0103 + 0.0175, *R*^2^ = 0.03) (Figure 6).

### 3.3. Field Experiment with Melon Flowers and Knapweed

**Figure 7:**
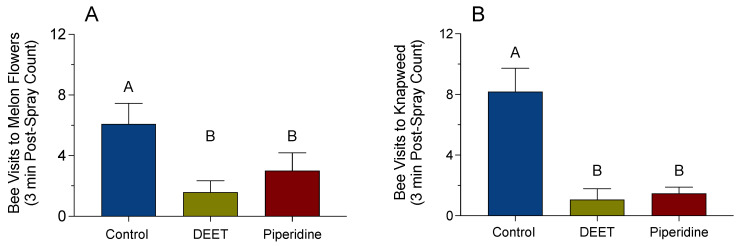
Effect of DEET and piperidine on the visitation of honey bees to melon flowers and knapweed bundles. Melon flowers (*N* = 240) and knapweed bundles (*N* = 51) were treated with deionized H_2_O (control), 10 µL/mL DEET, or 10 µL/mL piperidine. A 3 min post-spray count of visitation was performed and repeated 12 times in the melon plots and 17 times in the field of knapweed. (**A**) Mean number of honey bees and squash bees visiting the melon flowers post-spray (F_(2, 33)_ = 4.13, *p* < 0.02). (**B**) Mean number of honey bees visiting knapweed bundles post-spray (F_(2, 48)_ = 15.67, *p* < 0.0001). Vertical bars represent the mean ± standard error. Different letters above the bars indicate a significant difference using an ANCOVA with Student’s multiple comparison *t*-test.

The field experiments were conducted to determine whether DEET and piperidine can deter honey and squash bees from visiting melon flowers (Figure 7A) and honey bees from visiting knapweed (Figure 7B). There was no significant effect between the pre-treatment and post-treatment mean number of bees visiting the melon flowers (Figure 7A, *p* = 0.46). However, there was a significant reduction in the mean number of bees visiting the melon flowers treated with DEET and piperidine, when compared to the number of bees visiting the untreated melon flowers (Figure 7A). The mean number of bees visiting the melon flowers treated with DEET and piperidine was 1.6 ± 0.76 and 3 ± 1.2 bees per group of 20 treated flowers, respectively, compared to the 6.1 ± 1.4 bees per group of 20 untreated flowers. There was no significant difference between the mean numbers of bees visiting the melon flowers treated with DEET, when compared to the mean number of bees visiting the melon flowers treated with piperidine (Figure 7A).

Similarly, there was no significant effect between the pre-treatment and post-treatment number of bees visiting the knapweed (Figure 7B, *p* = 0.95). However, there was a significant reduction in the number of bees visiting the knapweed treated with DEET and piperidine, when compared to the number of bees visiting the untreated knapweed (Figure 7B). The number of bees visiting the knapweed treated with DEET and piperidine was 1.1 ± 0.71 and 1.5 ± 0.41 bees per treated bundle, respectively, compared to the 8.2 ± 1.5 bees per untreated bundle. There was no significant difference between the numbers of bees visiting the DEET-treated knapweed, when compared to the number of bees visiting the knapweed treated with piperidine (Figure 7B).

### 3.4. EAG Recordings

**Figure 8:**
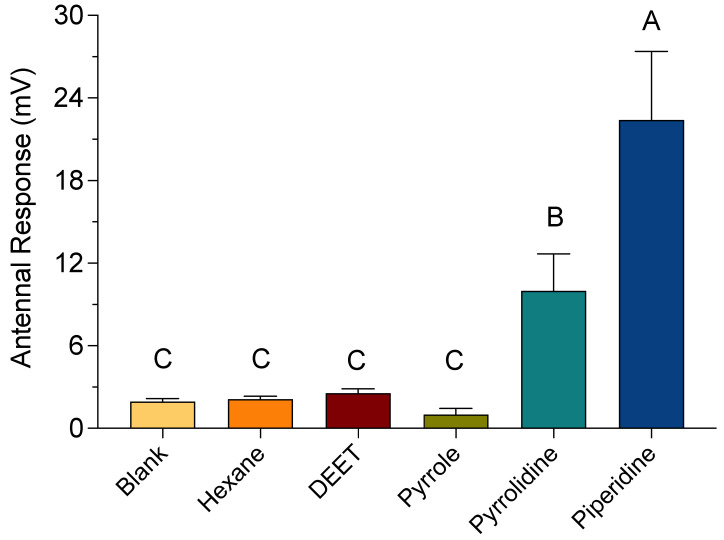
Electroantennogram (EAG) recordings of bee antennae exposed to heterocyclic amines (HCAs). The change in baseline voltage (mV) was calculated by subtracting the absolute value of the baseline voltage from the absolute value of the response voltage. An empty Pasteur pipette and hexane-treated filter paper served as a control (blank) and carrier solvent treatment, respectively. Bee antennae exposed to HCAs at 1000 µL/mL. Vertical bars represent the mean ± standard error. Different letters above the bars indicate a significant difference between treatments using an ANOVA with Tukey’s multiple comparison test (*p* < 0.05).

The changes in baseline voltages for bee antennae exposed to the HCAs at 10 µL/mL were neither significantly different from one another, nor were they significantly different from the control and solvent treatments. However, the HCAs at 1000 µL/mL produced significantly higher changes in baseline voltage compared to the control and solvent treatments (Figure 8). Baseline voltage changes for bee antennae exposed to piperidine, pyrrolidine, and pyrrole at 1000 µL/mL were 22.4 (± 5.0), 10.0 (± 2.7), 1.0 (± 0.5 SE) mV, respectively. A significantly greater response was observed for piperidine, when compared to pyrrolidine and pyrrole; however, the changes in baseline voltage for pyrrolidine and pyrrole were not significantly different from one another.

**Figure 9:**
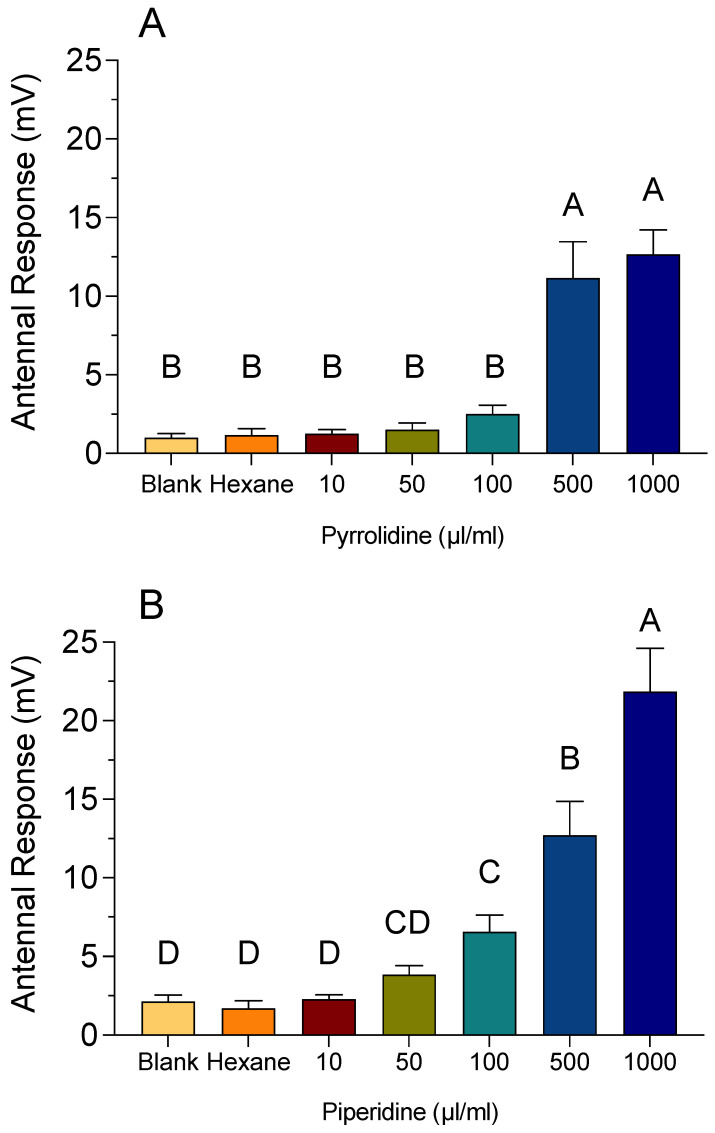
Electroantennogram (EAG) recordings of bee antennae exposed to the HCAs pyrrolidine (**A**) and piperidine (**B**). The change in voltage was calculated by subtracting the absolute value of the baseline voltage from the absolute value of the response voltage. An empty Pasteur pipette (blank) and hexane-treated filter paper served as a control and carrier solvent treatment, respectively. Vertical bars represent the mean ± standard error. Different letters above the bars indicate a significant difference between treatments using an ANOVA with Tukey’s multiple comparison test (*p* < 0.05).

EAG response amplitudes for bee antennae exposed to a concentration range of pyrrolidine and piperidine are shown in Figure 9. The changes in baseline voltages for bee antennae exposed to 10, 50, and 100 µL/mL of both pyrrolidine and piperidine were not significantly different from each other, nor were they significantly different from control and solvent treatments. However, the change in baseline voltages for bee antennae exposed to 500 and 1000 µL/mL pyrrolidine was 11.2 (±2.3) mV and 12.7 (±1.5) mV, respectively (Figure 9A). This was significantly different from control and solvent treatments, but not from each other. The changes in baseline voltages for bee antennae exposed to 500 and 1000 µL/mL piperidine were 12.7 (±2.2) mV and 22 (±2.6) mV, respectively, which were significantly different from each other and from the control and solvent treatments (Figure 9B).

## 4. Discussion

The inclusion of a repellent with pesticide applications has the potential to reduce the time pollinators are exposed to pesticides [12,30]. Numerous repellency studies have been focused on personal protection from disease-transmitting arthropods [13]. However, there are fewer studies reporting that pollinators can be repelled from areas that are sprayed with a chemical compound [12,30,31]. A total of seven piperazines, four piperidines, two pyrrolidines, and two pyrroles were observed to deter bees from a food source similar to DEET, when using video-tracking recordings. The efficacy of the HCA compounds was dependent on the substitutions of the parent ring structure. For pyrrolidine, efficacy was significantly increased with the addition of a methyl or ethyl group to the ring structure. Additions to the ring structure of piperazine also increased the efficacy of the parent compound. Both pyrrole and piperidine lose efficacy when substitutions are added to the ring structures. Taking these changes into consideration can aid in the development of more effective repellent compounds. For the discovery and development of repellents, there are several strategies that are used to increase the efficacy of lead compounds, such as structure–activity relationship analysis. This approach allows for the comparison of the molecular structure of the compounds to their physiochemical properties and biological responses [32]. An examination of additional structural arrangements of HCAs, along with concentration, efficacy, and longevity studies, in the sugar-agarose cubes and alternative HCA-infused food sources warrants further investigation to identify a more effective repellent for bees.

Piperidine was selected as the representative HCA to screen in the high-tunnel and field experiments not only because it was the most effective HCA observed using video-tracking recordings, but piperidine analogs have been reported to repel several types of arthropods. The analog 1-(3-cyclohexen-1-ylcarbonyl)-2-methylpiperidine was not only reported to repel the lone star tick, *Amblyomma americanum*, from humans significantly longer than DEET, but a stereoisomer of the analog was reported to be an effective repellent for mosquitoes and chigger mites [19,21]. Furthermore, the analog 1-(3-cyclohexenyl-carbonyl) piperidine was observed to provide protection against *A. americanum* 1.5 times longer than DEET [23]. The EPA currently recognizes seven active ingredients as topical insect repellents, including picaridin, which is a highly effective piperidine-based tick repellent [33]. The hypothesis that piperidine-based compounds will deter bees from a food source was based on the success of these compounds to repel other arthropods. In fact, this study demonstrates that piperidine does significantly deter bees from sugar feeders, melon flowers, and knapweed by reducing the visitation of foraging individuals to the feeders and flowers. A similar effect was observed with the number of bees visiting melon flowers and knapweed treated with DEET. The effects of DEET on forager bee visitation and recruitment to the feeder station was not examined due to the limited availability of nucleus bee colonies.

DEET has not only been observed to elicit a short-term inhibitory response of bee antennae to attractive volatiles [34], but also has been shown to be an effective gustatory repellent to bees when infused into a sugar solution [12]. Accordingly, it was observed in the present study that foraging bees would approach the DEET- and piperidine-treated melon flowers and flowering knapweed, but would leave before contacting the flowers. DEET has been observed to be less effective as an olfactory repellent than as a contact repellent [35,36], whereas there are reports that piperidine analogs appear to be contact repellents [19,21,23]. Bees exhibit tarsal gustation to not only sweet and saline solutions, but also gustation to bitter substances [37], such as DEET and piperidine analogs. Previous studies demonstrate DEET to activate bitter-tuned insect gustatory receptors [38], whereas picaridin, a piperidine analog, has been shown to elicit a response on bitter-sensitive insect gustatory receptors [39].

To better understand how the behavioral effects were being elicited, EAG recordings were conducted to determine whether the HCAs stimulated the bee olfactory system. The HCAs pyrrole, pyrrolidine, and piperidine at 10 µL/mL concentration did not appear to elicit an olfactory response by changing the baseline voltage of the bee antennae compared to that of the control and carrier solvent treatments. These HCAs were tested with a range of concentrations in an attempt to record an olfactory response of the bee antennae and, in fact, a change in the baseline voltage was observed with the bee antennae compared to that of the control and carrier solvent treatments. DEET did not elicit a significant olfactory response compared to the control and carrier solvent treatments. However, DEET has been shown to elicit a transient response with bee antennae [34], but at concentrations higher than those used for this study. It would be worthwhile to examine other substituted pyrroles, piperidines, and piperazines, at concentrations above 10 µL/mL, to not only differentiate the antennal sensitivities to each compound, but to construct and test hypotheses and contextualize the behavioral and antennal responses of bees.

Piperidine elicited the strongest EAG response in the bee antennae. This observation correlates to the results of the video-tracking experiments conducted previously. Pyrrolidine elicited an EAG response less than that of piperidine and pyrrole. Thus, pyrrolidine and pyrrole were tested against bee antennae in a concentration-dependent manner, with an EAG response detected at 100 µL/mL. These data suggest that the observed deterrence of bees from pyrrolidine- and pyrrole-treated food sources is an olfactory response.

## 5. Conclusions

This study provides evidence that HCAs elicit antennal responses of honey bees, which are, in turn, deterred from food sources. The method utilized here to record bee behavior is an excellent approach to determine whether a compound has a deterrent effect. However, this method lacks the ability to clearly discern repellency as the test arena is too small to view movement away from the chemical source. A strict definition of repellency states that an insect will make direct movement away from the chemical source [40]. Repellency has also been described, however, as a quality of a compound that will prevent an insect from reaching a target that it would otherwise find if the compound were not present [41]. Using this definition, an argument could be made that these compounds are repelling bees from the food cubes. The success of the HCAs warrants further study into the production of structure–activity relationships to develop more effective repellents. Screening additional HCA chemistries could lead to the discovery of a substituted version that is detected readily by the antennae. Additionally, further investigation into the sensory physiology of bees to decipher the behavioral effects observed could lead to discovering the mode of action of these compounds and, potentially, allow further investigation into receptor specificity. The bee genome encodes 170 olfactory genes in addition to 10 gustatory receptor genes [42,43,44]. Recent work has shown bee tarsal gustation to sweet, bitter, and saline substances [37]. Thus, we cannot exclude the potential for bees to respond to the taste of HCAs in addition to the observed olfactory responses, which would require additional experiments. Lastly, it should be noted that neither wild bee repellency and their pollination activity was studied with HCAs nor were the potential effects of these compounds to agricultural landscapes. This work is outside the scope of the current study, but would be complementary to on-going studies for the development of HCAs as short-term repellent compounds that are effective deterrents to reduce the contact of bees to pesticide-treated plants.

## Data Availability

The data presented in this study are available on request from the corresponding author.

## References

[1] Gallai N., Salles J.M., Settele J., Vaissiere B.E. (2009). Economic valuation of the vulnerability of world agriculture confronted with pollinator decline. Ecol. Econ..

[2] Mullin C.A., Frazier M., Frazier J.L., Ashcraft S., Simonds R., Pettis J.S. (2010). High levels of miticides and agrochemicals in North American apiaries: Implications for honey bee health. PLoS ONE.

[3] Van Engelsdorp D., Meixner M.D. (2010). A historical review of managed honey bee populations in Europe and the United States and the factors that may affect them. J. Invertebr. Pathol..

[4] Sanchez-Bayo F., Goka K. (2014). Pesticide residues and bees—A risk assessment. PLoS ONE.

[5] O’Neal S.T., Brewster C.C., Bloomquist J.R., Anderson T.D. (2017). Amitraz and its metabolite modulate honey bee cardiac function and tolerance to viral infection. J. Invertebr. Pathol..

[6] O’Neal S.T., Anderson T.D., Wu-Smart J.Y. (2018). Interactions between pesticides and pathogen susceptibility in honey bees. Curr. Opin. Insect Sci..

[7] Reeves A.M., O’Neal S.T., Fell R.D., Brewster C.C., Anderson T.D. (2018). In-hive acaricides alter biochemical and morphological indicators of honey bee nutrition, immunity, and development. J. Insect Sci..

[8] O’Neal S.T., Reeves A.M., Fell R.D., Brewster C.C., Anderson T.D. (2019). Chlorothalonil exposure alters virus susceptibility and markers of immunity, nutrition, and development in honey bees. J. Insect Sci..

[9] Carvalho F.P. (2017). Pesticides, environment, and food safety. Food Energy Secur..

[10] Jones G.D.G. (1952). The responses of the honey-bee to repellent chemicals. J. Exp. Biol..

[11] Woodrow A.W., Green N., Tucker H., Schonhorst M.H., Hamilton K.C. (1965). Evaluation of chemicals as honey bee attractants and repellents. J. Econ. Entomol..

[12] Atkins E.L., Macdonald R.L., Greywood-Hale E.A. (1975). Repellent additives to reduce pesticide hazards to honey bees: Field tests. Environ. Entomol..

[13] Katz T.M., Miller J.H., Hebert A.A. (2008). Insect repellents: Historical perspectives and new developments. J. Am. Acad. Dermatol..

[14] Brown M., Hebert A.A. (1997). Insect repellents: An overview. J. Am. Acad. Dermatol..

[15] Dickens J.C., Bohbot J.D. (2013). Mini review: Mode of action of mosquito repellents. Pestic. Biochem. Phys..

[16] Lawrence K.L., Achee N.L., Bernier U.R., Mundal K.D., Benante J.P. (2014). Field evaluations of topical arthropod repellents in North, Central, and South America. J. Med. Entomol..

[17] Diaz J.H. (2016). Chemical and plant-based insect repellents: Efficacy, safety, and toxicity. Wilderness Environ. Med..

[18] McGovern T.P., Schreck C.E., Jackson J. (1978). Mosquito repellents: Alicyclic amides as repellents for *Aedes aegypti* and *Anopheles quadrimaculatus*. Mosq. News.

[19] Solberg V.B., Klein T.A., McPherson K.R., Bradford B.A., Burge J.R., Wirtz R.A. (1995). Field evaluation of DEET and a piperidine repellent (Ai3-37220) against *Amblyomma americanum* (Acari: Ixodidae). J. Med. Entomol..

[20] Debboun M., Strickman D., Solberg V.B., Wilkerson R.C., McPherson K.R., Golenda C., Keep L., Wirtz R.A., Burge R., Klein T.A. (2000). Field evaluation of DEET and a piperidine repellent against *Aedes communis* (Diptera: Culicidae) and *Simulium venustum* (Diptera: Simuliidae) in the Adirondack Mountains of New York. J. Med. Entomol..

[21] Klun J.A., Schmidt W.F., Debboun M. (2001). Stereochemical effects in an insect repellent. J. Med. Entomol..

[22] Klun J.A., Khrimian A., Margaryan A., Kramer M., Debboun M. (2003). Synthesis and repellent efficacy of a new chiral piperidine analog: Comparison with DEET and Bayrepel activity in human-volunteer laboratory assays against *Aedes aegypti* and *Anopheles stephensi*. J. Med. Entomol..

[23] Schreck C.E., Fish D., McGovern T.P. (1995). Activity of repellents applied to skin for protection against *Amblyomma americanum* and *Ixodes scapularis* ticks (Acari: Ixodidae). J. Am. Mosq. Control. Assoc..

[24] Klun J.A., Schmidt W.F. (2003). Methods and Compositions for Repelling Arthropods.

[25] Yang L., Liu Y., Bernier U., Tsikolia M., Linthicum K., Bloomquist J. (2018). Vapor Phase Repellents: New Methods, Chemistry, and Mechanisms of Action.

[26] Larson N.R., Anderson T.D. (2017). Video tracking protocol to screen deterrent chemistries for honey bees. JoVE.

[27] Wilson J.M., Anderson T.D., Kuhar T.P. (2019). Sublethal effects of the insecticide pyrifluquinazon on the European honey bee (Hymenoptera: Apidae). J. Econ. Entomol..

[28] Tangtrakulwanich K., Chen H., Baxendale F., Brewer G., Zhu J.J. (2011). Characterization of olfactory sensilla of *Stomoxys calcitrans* and electrophysiological responses to odorant compounds associated with hosts and oviposition media. Med. Vet. Entomol..

[29] Larson N.R., O’Neal S.T., Bernier U.R., Bloomquist J.R., Anderson T.D. (2020). Terpenoid-induced feeding deterrence and antennal response of honey bees. Insects.

[30] Free J.B., Pickett J.A., Ferguson A.W., Simpkins J.R., Smith M.C. (1985). Repelling foraging honeybees with alarm pheromones. J. Agric. Sci..

[31] Collins A.M., Rubink W.L., Cuadriello Aguilar J.I., Hellmich R.L. (1996). Use of insect repellents for dispersing defending honey bees (Hymenoptera: Apidae). J. Econ. Entomol..

[32] Das S. (2016). Screening of bioactive compounds for development of new pesticides: A mini review. Univers. J. Agric. Res..

[33] Carroll J.F., Benante J.P., Klun J.A., White C.E., Debboun M., Pound J.M., Dheranetra W. (2008). Twelve-hour duration testing of cream formulations of three repellents against *Amblyomma americanum*. Med. Vet. Entomol..

[34] Singh N.K., Eliash N., Kamer Y., Zaidman I., Plettner E., Soroker V. (2015). The effect of DEET on chemosensing of the honey bee and its parasite *Varroa destructor*. Apidologie.

[35] Bernier U.R., Furman K.D., Kline D.L., Allan S.A., Barnard D.R. (2005). Comparison of contact and spatial repellency of catnip oil and N,N-diethyl-3-methylbenzamide (deet) against mosquitoes. J. Med. Entomol..

[36] Abramson C.I., Giray T., Mixson T.A., Nolf S.L., Wells H., Kence A., Kence M. (2010). Proboscis conditioning experiments with honeybees, *Apis mellifera caucasica*, with butyric acid and DEET mixture as conditioned and unconditioned stimuli. J. Insect Sci..

[37] De Brito Sanchez M.G., Lorenzo E., Su S., Liu F., Zhan Y., Giurfa M. (2014). The tarsal taste of honey bees: Behavioral and electrophysiological analyses. Front. Behav. Neurosci..

[38] Sanford J.L., Shields V.D.C., Dickens J.C. (2013). Gustatory receptor neuron responds to DEET and other insect repellents in the yellow-fever mosquito, *Aedes aegypti*. Naturwissenschaften.

[39] Sparks J.T., Dickens J.C. (2016). Bitter-sensitive gustatory receptor neuron responds to chemically diverse insect repellents in the common malaria mosquito *Anopheles quadrimaculatus*. Sci. Nat..

[40] Dethier V.G., Browne B.L., Smith C.N. (1960). The designation of chemicals in terms of the responses they elicit from insects. J. Econ. Entomol..

[41] Bernier U., Kline D., Posey K.H., Mustapha Debboun S.F., Strickman D. (2006). Human emanations and related natural compounds that inhibit mosquito host-finding abilities. Insect Repellents: Principles, Methods and Uses.

[42] Robertson H.M., Wanner K.W. (2006). The chemoreceptor superfamily in the honey bee, *Apis mellifera*: Expansion of the odorant, but not gustatory, receptor family. Genome Res..

[43] Dunipace L., Meister S., McNealy C., Amrein H. (2001). Spatially restricted expression of candidate taste receptors in the *Drosophila* gustatory system. Curr. Biol..

[44] Scott K., Brady R., Cravchik A., Morozov P., Rzhetsky A., Zuker C., Axel R. (2001). A chemosensory gene family encoding candidate gustatory and olfactory receptors in *Drosophila*. Cell.

